# A new peucemycin derivative and impacts of *peuR* and *bldA* on peucemycin biosynthesis in *Streptomyces peucetius*

**DOI:** 10.1007/s00253-023-12923-4

**Published:** 2024-01-12

**Authors:** Rubin Thapa Magar, Van Thuy Thi Pham, Purna Bahadur Poudel, Adzemye Fovennso Bridget, Jae Kyung Sohng

**Affiliations:** 1 Department of Life Science and Biochemical Engineering, Sun Moon University, 70 Sun Moon-Ro 221, Tangjeong-Myeon, Asan-Si, 31460 Chungnam Korea; 2 Department of Pharmaceutical Engineering and Biotechnology, Sun Moon University, 70 Sun Moon-Ro 221, Tangjeong-Myeon, Asan-Si, 31460 Chungnam Korea

**Keywords:** *Streptomyces peucetius*, Peucemycin, 19-hydroxy peucemycin, SARP regulator, Global regulator

## Abstract

**Abstract:**

*Streptomyces peucetius* ATCC 27952 is known to produce a variety of secondary metabolites, including two important antitumor anthracyclines: daunorubicin and doxorubicin. Identification of peucemycin and 25-hydroxy peucemycin (peucemycin A), as well as their biosynthetic pathway, has expanded its biosynthetic potential. In this study, we isolated a new peucemycin derivative and identified it as 19-hydroxy peucemycin (peucemycin B). Its antibacterial activity was lower than those of peucemycin and peucemycin A. On the other hand, this newly identified peucemycin derivative had higher anticancer activity than the other two compounds for MKN45, NCI-H1650, and MDA-MB-231 cancer cell lines with IC_50_ values of 76.97 µM, 99.68 µM, and 135.2 µM, respectively. Peucemycin biosynthetic gene cluster revealed the presence of a SARP regulator named PeuR whose role was unknown. The presence of the TTA codon in the *peuR* and the absence of global regulator BldA in *S. peucetius* reduced its ability to regulate the peucemycin biosynthetic gene cluster. Hence, different mutants harboring these genes were prepared. *S. peucetius* bldA25 harboring *bldA* produced 1.75 times and 1.77 times more peucemycin A (11.8 mg/L) and peucemycin B (21.2 mg/L), respectively, than the wild type. On the other hand, *S. peucetius* R25 harboring *peuR* produced 1.86 and 1.79 times more peucemycin A (12.5 mg/L) and peucemycin B (21.5 mg/L), respectively, than the wild type. Finally, strain *S. peucetius* bldAR25 carrying *bldA* and *peuR* produced roughly 3.52 and 2.63 times more peucemycin A (23.8 mg/L) and peucemycin B (31.5 mg/L), respectively, than the wild type.

**Key points:**

*• This study identifies a new peucemycin derivative*, *19-hydroxy peucemycin* (*peucemycin B*).

*• The SARP regulator* (*PeuR*)* acts as a positive regulator of the peucemycin biosynthetic gene cluster*.

• *The overexpression of peuR and heterologous expression of bldA increase the production of peucemycin derivatives*.

**Supplementary Information:**

The online version contains supplementary material available at 10.1007/s00253-023-12923-4.

## Introduction

*Streptomyces peucetius* produces a number of bioactive secondary metabolites including daunorubicin (DNR), doxorubicin (DXR) (Arcamone and Cassinelli 1998; Arcamone et al. 1969), flaviolin (Ghimire et al. 2008), geosmin (Singh et al. 2009), peucechelin (Park et al. 2013) (Kodani et al. 2015), and hopene (Ghimire et al. 2015). Furthermore, a new 14-membered macrolide (peucemycin) with a γ-pyrone ring was purified and assigned its structure (Pham et al. 2021). The compound exhibited antibacterial activities against some pathogens and suppressed the viability of various cancer cell lines (Pham et al. 2021). Recently, a new peucemycin derivative and its putative biosynthetic gene cluster were identified, providing insight into the biosynthesis of peucemycin and its derivatives (Magar et al. 2023). The newly identified derivative was 25-hydroxy peucemycin (peucemycin A) and had antibacterial activities against some pathogens (Magar et al. 2023).

The peucemycin biosynthetic gene cluster (Peu BGC) has two cluster-situated regulators (pathway-specific regulators). Among the two, PeuR was identified as a *Streptomyces* antibiotic regulator protein (SARP) regulator having a helix-turn-helix (HTH) DNA-binding domain motif at the N-terminal and a bacterial transcriptional activator domain (BTAD) at their C terminus (Hindra and Elliot 2010; Magar et al. 2023). It is currently unknown how PeuR controls the production of peucemycin and its derivatives.

The regulation of biosynthetic pathways by regulatory genes functions as pathway-specific regulators (also known as cluster-situated regulators) and/or pleiotropic (or global) regulators (Lu et al. 2017). Some biosynthetic gene clusters necessitate the participation of both regulators, such as pathway-specific regulatory gene *actII-*ORF4 and global regulatory gene *cprB* (Fernández-Moreno et al. 1991) in the production of actinorhodin (ACT) from *S. coelicolor* (Onaka et al. 1998). Similarly, the production of polyketide can be improved by controlling the regulatory mechanism (Chen et al. 2010). For instance, overexpression of SARP regulator in *Streptomyces* sp. KCCM11116P resulted in a higher production titer of tacrolimus (FK506) compared to the wild type (Chen et al. 2015). Furthermore, overexpression of the SARP regulator can activate a silent gene cluster. One example is the activation of the undecylprodigiosin (Red) gene cluster by the overexpression of the *papR2* from *S. pristinaespiralis* in *S. lividans* (Krause et al. 2020).

In addition to the pathway-specific regulator, a global regulator BldA also participates in the regulation of biosynthetic pathways. The *bldA* encodes tRNA required for the translation of a rare UUA codon for leucine (Chater and Chandra 2008). Hence, the BldA affects the translation of genes having this rare codon. It also plays an important role in the regulation of antibiotic production (Hackl and Bechthold 2015) like undecylprodigiosin (RED) biosynthesis in *S. coelicolor* (White and Bibb 1997) and landomycin E in *S. globisporus* 1912 (Rebets et al. 2006).

In this study, we successfully isolated another peucemycin derivative and determined its structure. The impact of overexpressing *peuR*, a SARP regulator, on biosynthesis of peucemycin and its derivatives was also thoroughly investigated. Furthermore, the effect of heterologous expression of *bldA*, a pleiotropic regulator, on biosynthesis of peucemycin and its derivatives was investigated.

## Materials and methods

### Bacterial strains, media, and culture conditions

*Escherichia coli* XL1-Blue MRF (Stratagene) was used as the cloning host, while *E. coli* ET12567 (John Innes Centre, UK) was used as the demethylating host. They were cultured in Luria–Bertani (LB) media at 37 °C with ampicillin (100 µg/mL) when necessary. For cloning PCR fragments, pGEM®–T Easy vector (Promega) was used. As an expression vector for *S. peucetius*, pIBR25 (Sthapit et al. 2004) was used. Standard protocols were used for manipulating DNA in *E. coli* (Sambrook and Russell 2001). *S. peucetius* and its derived strains were grown in R2YE media at 28 °C with thiostrepton (15 µg/mL) when necessary. Their protoplasts were prepared in tryptic soya broth (TSB) following the standard protocol (Kieser et al. 2000). Peucemycin and its derivatives were produced by growing *S. peucetius* strains in Hickey-Tresner (HT) media at 18 °C for 3 days (Hickey and Tresner 1952). All wild-type and recombinant strains along with vectors developed during this study are listed in Table S1.

### Construction of recombinant vectors and generation of mutant strains

Standard protocols were followed for DNA manipulations and the production of mutants (Kieser et al. 2000; Sambrook and Russell 2001). The *peuR* and *bldA* were amplified from genomic DNAs of *S. peucetius* DM07 and *S. coelicolor* A3, respectively, using primer pair listed in Table S2. Conditions for PCR amplification were 95 °C for 7 min; by 30 cycles of 95 °C for 30 s, 55–68 °C for 30 s, 72 °C for 60–90 s; and final extension at 72 °C for 7 min. DNA amplification was performed using PrimeSTAR HS DNA Polymerase (Takara) according to the manufacturer’s instructions. Following ligation into the pGEM-T Easy vector, sequences were verified.

After *peuR* and *bldA* were ligated separately into pIBR25 at *Xb*aI-*Hin*dIII and *Bam*HI-*Xba*I sites, respectively, the combination of both genes was produced using *Bam*HI-*Hin*dIII sites of pIBR25. All recombinant vectors were demethylated by *E. coli* ET12567 and subsequently transferred into *S. peucetius* using polyethylene glycol (PEG)-mediated protoplast transformation (Malla et al. 2009). Transformants were selected using R2YE plates with thiostrepton. Confirmation of the transformation was done by isolating recombinant vectors from positive clones that were resistant to thiostrepton. A single colony from each mutant was taken to study the production of peucemycin and its derivatives.

### Analysis of growth rate and production of peucemycin and its derivatives

To determine the optimal time for producing 19-hydroxypeucemycin (peucemycin B), a seed culture was prepared by growing *S. peucetius* DM07 in R2YE media for 48 h at 28 °C. Then 1 mL seed culture was added to 50 mL HT media and cultured for 168 h at 18 °C. At every 24-h time interval, 1 mL of culture broth was collected for 168 h. The remaining culture broths were used for quantification of peucemycin B. Samples were centrifuged at 12,000 rpm and washed twice with sterile distilled water. Pellets were finally dried at 70 °C for 4 days, and dry cell weight (DCW) for each sample was measured. Measurements were taken for three biological replicates. Peucemycin B was quantified after the remaining culture broths were extracted with ethyl acetate at double volume. The organic phase was dried using a rotary evaporator under reduced pressure. Each extract was then dissolved in 1 mL of methanol.

Samples were analyzed by Thermo HPLC series 1100 with a Thermo-C_18_ column (5 µm, 4.6 mm × 250 mm) which was equilibrated with 90% solvent A (water + 0.1% trifluoroactetic acid (TFA)) and 10% acetonitrile (ACN). The condition for analysis was a linear gradient of 1–25 min with 10% to 90% B, 25–28 min with 90% to 50% B, and 28 − 30 min with 50% to 10% B at a flow rate of 1 mL/min with UV detection at 268 nm. A standard curve was prepared with different concentrations of peucemycin B. Concentration of peucemycin B was calculated based on the peak area. Compound mass was analyzed by ultra-high-performance liquid chromatography electrospray ionization quadrupole time-of-flight high-resolution mass spectrometry (UPLC − ESI − Q − TOF − HRMS) using an Acquity column (UPLC; Waters Corp., Milford, MA, USA) coupled with a SYNAPT G2-S (Waters Corp) with a gradient of solvent mobile phase of 0.1% TFA in water and 100% ACN (0 to 12 min) at 35 °C. The sample volume used for injection was 10 µL. LC − MS analysis was performed using a high-resolution mass spectrometer equipped with an electrospray ionization source under the following conditions: 3 kV of capillary voltage, 300 °C of desolvation gas temperature, and 600 L/h of desolvation gas flow rate.

Growth rate and peucemycin production for all other mutants were studied by taking samples for 3 days and performing similar steps as mentioned above. Calibration curves were prepared for peucemycin and peucemycin A. All the data were generated from three experimental samples, and a student *t*-test was performed with GraphPad Prism 6 for significance tests.

### Isolation and purification of peucemycin B

For sample preparation, *S. peucetius* DM07 was grown in HT media for 72 h at 18 °C in a 4 L fermenter. A total of 12 L of HT medium was used to isolate peucemycin B. Double volume of ethyl acetate was added to the sample and the organic phase was dried using a rotary evaporator under reduced pressure. This crude extract was washed twice with autoclaved distilled water followed by washing once with hexane. The sample was then washed with dichloromethane and dried. The final extract was dissolved in 150 mL of methanol. The extract was filtered and purified using Dionex Ultimate 3000 UPLC (Thermo Fisher Scientific) with a C_18_ column (YMC − Pack ODS − Aq, 150 × 20 mm^2^) connected to a UV detector (220 and 268 nm). The condition for binary gradient of mobile phase was 100% water (solvent A) and 100% acetonitrile (solvent B) (0–5 min, 0 to 20% B; 5–10 min, 20 to 40% B, 10–20 min, 40 to 60% B; 20–28 min, 60 to 100% B; 28–30 min, 100% B; and 30–35 min, 100 to 0% B) at a flow rate of 10 mL/min. The purified compound was dried, lyophilized, and dissolved in dimethyl sulfoxide (DMSO-*d*_*6*_), and then subjected to 700 MHz analyses using a Bruker BioSpin nuclear magnetic resonance (NMR) spectrometer (Billerica, USA), including one-dimensional (1D) ^1^H- NMR,^13^C-NMR, two-dimensional (2D) correlated spectroscopy (COSY), rotational frame nuclear overhauser effect (NOE) spectroscopy (ROESY), heteronuclear single quantum correlation (HSQC) analysis, and heteronuclear multiple bond correlation (HMBC) analysis (Ochang, Republic of Korea).

### Transcriptional level analysis

Total RNAs were isolated from 48 h bacterial cultures using an RNeasy Mini Kit (Qiagen Inc., USA) following the manufacturer’s protocols. DNA contamination was removed using RNase-Free DNase (Qiagen Inc., USA). Total RNA concentration and purity were determined using a spectrophotometer at 260 nm. Finally, cDNA was prepared by QuantiTect® Reverse Transcription kit (Qiagen) with an equal amount of RNA. RT-PCR conditions were cDNA synthesis at 50 °C for 30 min; initial denaturation at 95 °C for 10 min followed by 45 cycles of denaturation for 1 min at 95 °C, annealing at 1 min for 64 °C, and elongation at 72 °C for 1 min for PCR. Finally, PCR products were visualized using 1% agarose gel. Primers used for this study are listed in Table S2.

### Biological assays

The paper disk method was used to evaluate the antibacterial activities of peucemycin B against Gram-positive bacteria including both MRSA and MSSA strains of *Staphylococcus aureus* and Gram-negative bacteria *Proteus hauseri* NBRC 3851. The compound dissolved in DMSO was used for the test along with DMSO as a negative control and erythromycin as a positive control. The antibacterial effect was assessed after incubating plates at 37 °C for 16 − 24 h (Weinstein et al. 2018; Poudel et al. 2022).

Five different human cancer cell lines were obtained from the Korean Cell Line Bank (Seoul, Korea). MKN45 gastric cancer and NCI-H1650 lung cancer cell lines were grown in RPMI-1640 media (Corning Cellgro, Manassas, VA, USA) supplemented with 10% fetal bovine serum (FBS; R&D systems, Minneapolis, MN, USA). Hep3B liver cancer cells, MDA-MB-231 breast cancer cells, and MRC-5 lung normal cells were grown in DMEM medium (Corning Cellgro) supplemented with 10% FBS. U87MG brain cancer cell line was cultured with MEM medium (Corning Cellgro) supplemented with 10% FBS. All cells were cultured at 37 °C in a humidified CO_2_ incubator with 5% CO_2_ (Thermo Fisher Scientific, Vantaa, Finland). For cell viability assay, a CellTiter-Glo® luminescent assay system (Promega, Madison, WI, USA) was used according to the manufacturer’s instructions. Briefly, cells were seeded into a 96-white well culture plate at a density of 3 × 10^3^ cells/well and treated with peucemycin or its derivative at 0–400 µM for 72 h. After adding 20 µL of substrate solution to each well, culture plates were shaken for 2 min. They were then incubated at room temperature in the dark for 10 min. Luminescence was measured using a multimode microplate reader. Finally, IC_50_ values were determined with acquired data utilizing GraphPad Prism 6.

## Results

### Identification of a new peucemycin derivative

**Fig. 1:**
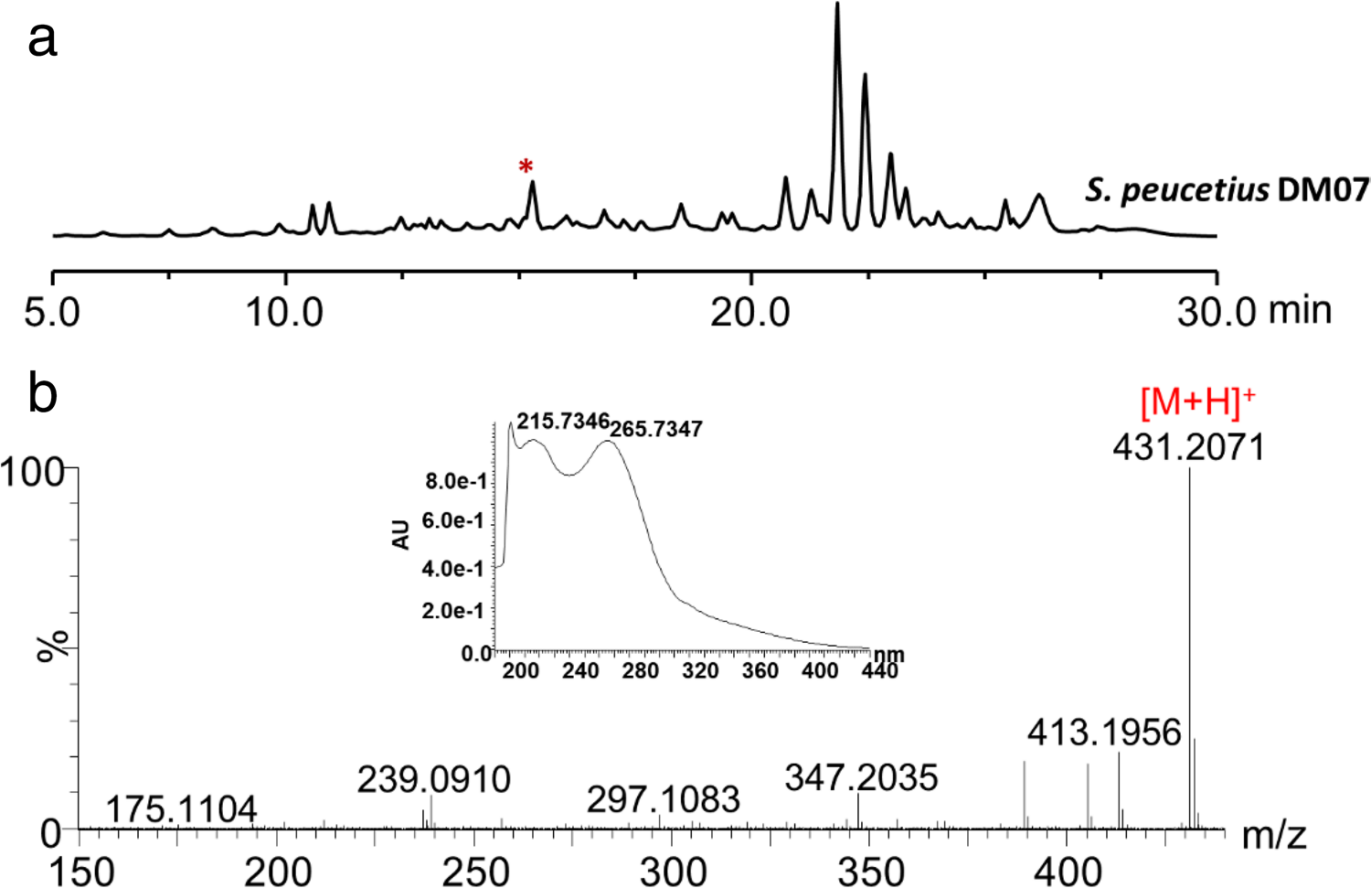
HPLC and mass analyses of the culture extract of *S. peucetius* DM07. **a** HPLC profile of culture extract, red asterisk represents the probable peucemycin derivative. **b** UV absorption and HRMS spectrum of peucemycin B

Mass analysis and UV absorption pattern for the peak at 15.31 min indicated that it was likely a derivative of peucemycin (Fig. 1). The structure was supported by LC–MS/MS analysis of the compound with *m/*z 431.2071 [M + H]^+^ (expected mass *m/*z 431.2064), *m/*z 413.1956 [M-OH + H]^+^ (expected mass *m/*z 413.1959), *m/*z 175.1104 [M-B + H]^+^ (expected mass *m/*z 175.1117), and *m/*z 239.0910 [M-A + H]^+^ (expected mass *m/*z 239.0914) (Fig. S1). These findings led to the conclusion that the newly discovered compound was hydroxylated peucemycin. Furthermore, the compound had absorbance maxima of 200.7346, 215.7346, and 265.7347 nm similar to peucemycin and peucemycin A (Magar et al. 2023; Pham et al. 2021) (Fig. 1).

### Growth rate analysis and production of peucemycin B

The maximum peucemycin B production occurred at 72 h, while the maximum growth occurred at 144 h (Fig. S2). At 72 h, the yield of peucemycin B was found to be 11.48 mg/L. Therefore, *S. peucetius* DM07 was cultured for 72 h to isolate the compound and determine its structure.

### Structure elucidation of peucemycin B

**Fig. 2:**
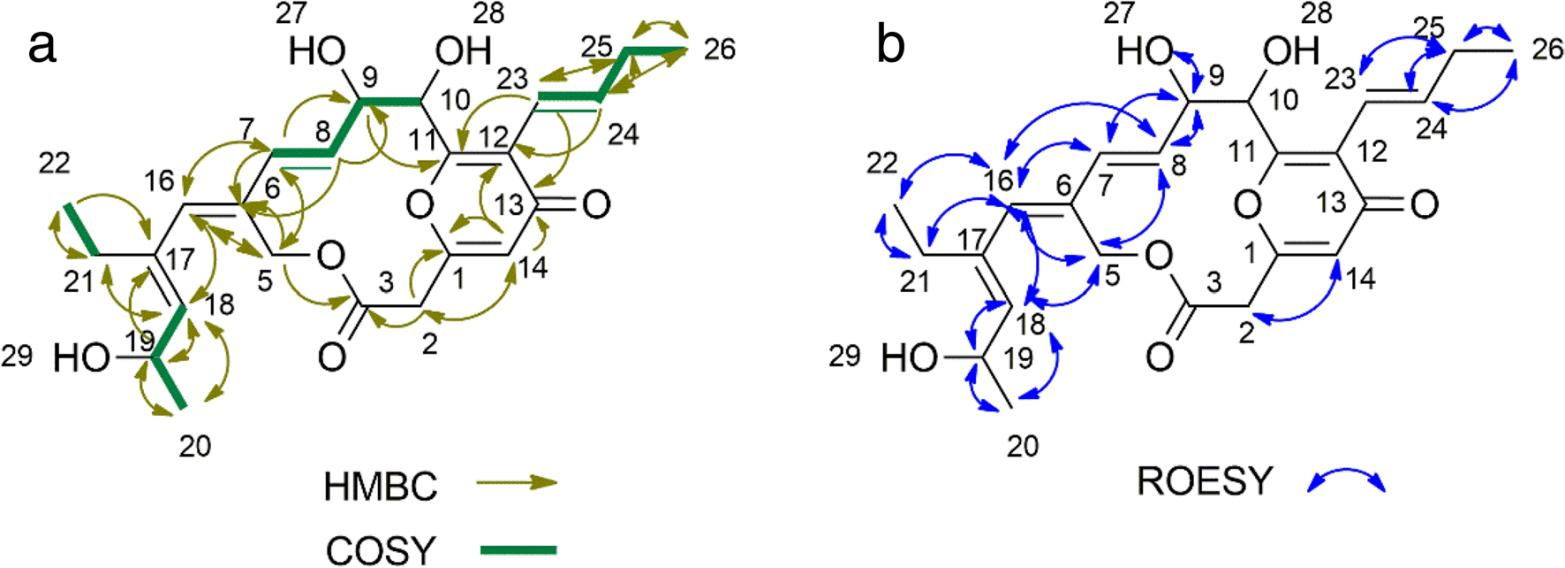
Two-dimensional NMR correlation of peucemycin B. **a** ^1^H-^1^H COSY (green bold line) and HMBC (arrow). **b** ^1^H-^1^H ROESY (blue arrow)

The compound was isolated as a yellow powder that could readily dissolve in dimethylsulfoxide (DMSO). The structure of the compound was examined using NMR spectroscopy. Results are summarized in Table 1, Fig. 2, and Fig. S3. Compared to the structure of peucemycin (Pham et al. 2021), a chemical shift (δ_C_) of C-19 from 21.24 ppm of peucemycin to 62.89 ppm of peucemycin B was found. Similarly, a chemical shift (δ_C_) of C-20 from 14.01 ppm of peucemycin to 24.35 ppm of peucemycin B was found. 2D NMR data further supported the structure of peucemycin B. Correlation between carbon and proton of peucemycin B is summarized in Fig. 2. COSY correlations were observed between H-25/Me-26, H23/H24, H-19/Me-20, H-21/Me-22, and H-19/H-18 (Fig. S3c). Similarly, HMBC correlations were found between Me-20 to C-19, 18, Me-22 to C-21, C-17, and Me-26 to C-25, C24 (Fig. S3f). These results revealed the existence of three methyl groups in the structure. The presence of methine groups was identified from COSY correlations as seen among H-7 and H-8, and H-23 and H-24 while similar HMBC correlations were seen among H-23 and H-24 to C-25 and, H-7 and H-8 to H-9 and H-6. ROESY correlations among protons of C-9, C-8, and C-7 revealed that they were all present on the same side. Figure S3d gives information on additionally observed correlations. All of these results supported that the structure was 19-hydroxy peucemycin (peucemycin B).

**Table 1:** ^1^H- and ^13^C-NMR data of peucemycin B in DMSO-*d*_6_

No	δ_C_, type	δ_H_, (*J*, Hz)	No	δ_C_, type	δ_H_, (*J*, Hz)
1	160.25, C		14	114.46, CH	6.21 (1 H, s)
2	39.17, CH_2_	3.65 (1 H, d, *J* 15.8) 3.73 (1 H, d, *J* 15.8)	16	136.17, CH	6.06 (1 H, s)
3	167.33, CO		17	131.71, C	
5	60.70, CH_2_	5.00 (1 H, d, *J* 11.9) 5.04 (1 H, d, *J* 12.0)	18	135.84, CH	5.12 (1 H, d, *J* 8.2)
6	133.28, C		19	62.89, CH	4.43 (1 H, m)
7	129.26, CH	6.02 (1 H, d, *J* 15.8)	20	24.35, CH_3_	1.12 (3 H, d, *J* 6.0)
8	130.65, CH	5.71 (1 H, dd, *J* 15.6, 4.9)	21	23.79, CH_2_	2.04 (2 H, m)
9	74.54, CH	4.33 (1 H, d, *J* 4.8)	22	13.54, CH_3_	0.90 (3 H, t, *J* 7.4)
10	72.14, CH	4.92 (1 H, s)	23	118.82, CH	6.27 (1 H, d, *J* 16.4)
11	162.89, C		24	138.65, CH	6.81 (1 H, dt, *J* 13.87, 6.34, 6.34)
12	122.54, C		25	26.79, CH_2_	2.11 (2 H, m)
13	178.41, CO		26	13.66, CH_3_	1.00 (3 H, t, *J* 7.4)

## Biological activities

Antibacterial activities of peucemycin B were weaker than those of peucemycin and peucemycin A. For *Proteus hauseri* NBRC 3851, peucemycin produced about two times higher antibacterial effect than peucemycin B when equivalent amounts of compounds were used (Fig. S4b). The growth of some *S. aureus* strains like *S. aureus* CCARM 0204 (MSSA) and *S. aureus* CCARM 3090 (MRSA) was inhibited by the compound at a high amount (Fig. S4a). These data showed that the compound had less antibacterial effects than peucemycin and peucemycin A.

**Fig. 3:**
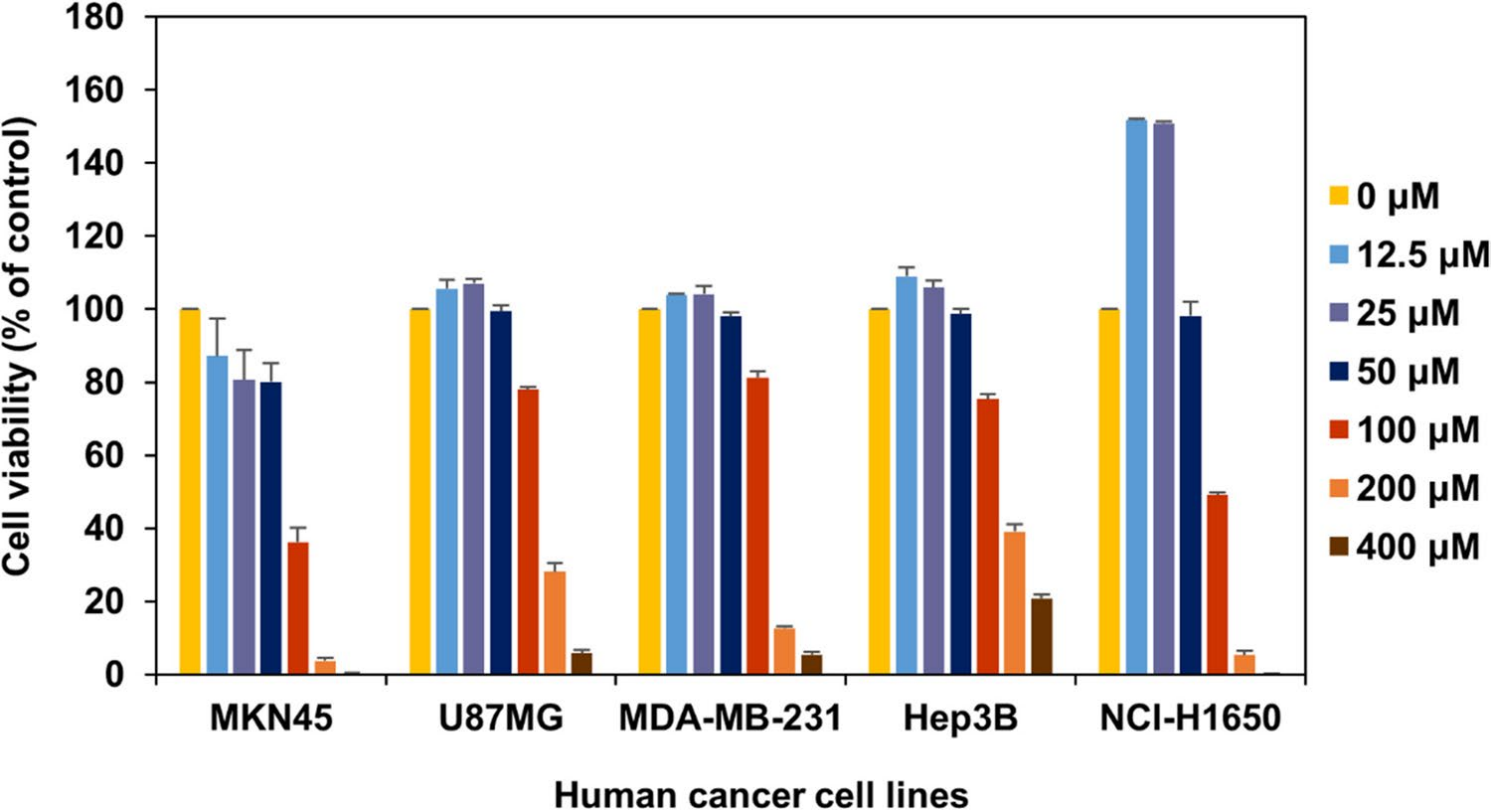
Anticancer activities of peucemycin B for various cancer cell lines. Standard deviation was calculated and represented as error bars from three experimental replicates for each sample

Five different cancer cell lines were used to evaluate the anticancer activities of the compound (Fig. 3). IC_50_ values for MKN45 and NCI-H1650 were found to be 76.97 µM and 99.68 µM, respectively (Table S3). These results showed that the compound has mild activity against these cell lines. Similarly, for three other cell lines MDA-MB-231, U87MG, and Hep3B, its IC50 values were found to be 135.2 µM, 150.0 µM, and 175.4 µM, respectively (Table S3). These results showed that the compound was weakly active against these cell lines.

### Heterologous expression of *bldA* and overexpression of *peuR*

The nucleotide sequence of *peuR* was found to have three rare TTA codons at the 13th, 52nd, and 74th codon positions that code for leucine (Fig. S6). *S. peucetius* does not have tRNA for this codon. Hence, *bldA* from *S. coelicolor* was heterologously expressed along with overexpression of *peuR*. At first, 873 bp *bldA* was amplified from *S. coelicolor* and ligated into pIBR25 at *Bam*HI and *Xba*I sites to generate pbldA25 (Fig. S7). Then, 963 bp *peuR* was amplified from *S. peucetius* DM07 and ligated into pIBR25 at *Xba*I and *Hin*dIII sites to generate pR25 (Fig. S7). Finally, pbldAR25 vector having both genes was constructed by ligating *peuR* at *Xba*I and *Hin*dIII sites of pbldA25. The observation of a fragment with a size of 1836 bp in the gel after digesting with *Bam*HI and *Hin*dIII confirmed the presence of both genes in pIBR25. Transformation of these vectors resulted in mutants *S. peucetius* R25, *S. peucetius* bldA25, *S. peucetius* bldAR25, and *S. peucetius* P25.

### Analysis of the production of peucemycin and its derivatives as well as growth rate

**Fig. 4:**
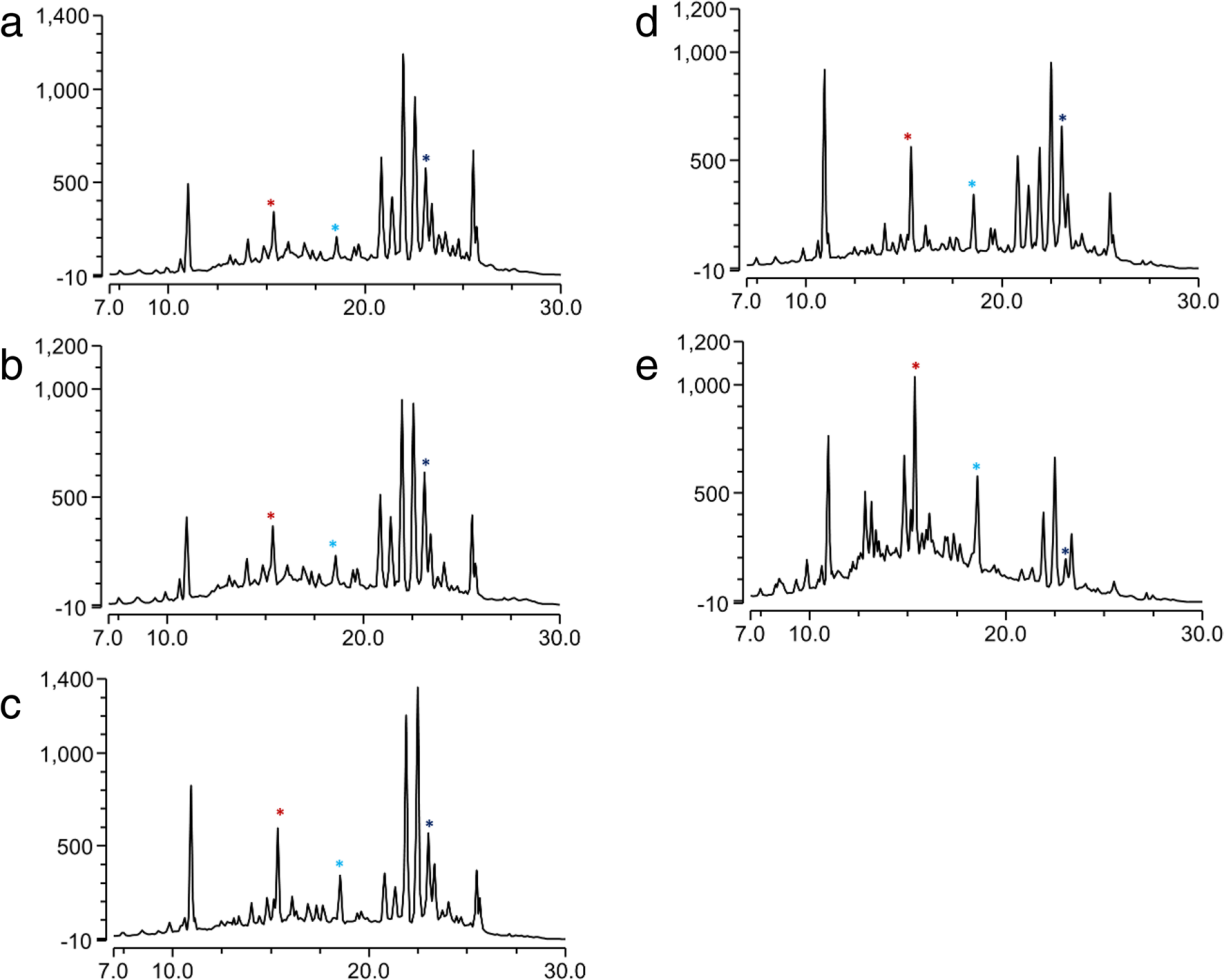
HPLC profiles of culture extracts. **a** *S. peucetius* DM07, **b** *S. peucetius* P25, **c** *S. peucetius* bldA25, **d** *S. peucetius* R25, and **e** *S. peucetius* bldAR25. Dark blue asterisk is for peucemycin. Sky blue asterisk is for peucemycin A. Red asterisk is for peucemycin B

**Fig. 5:**
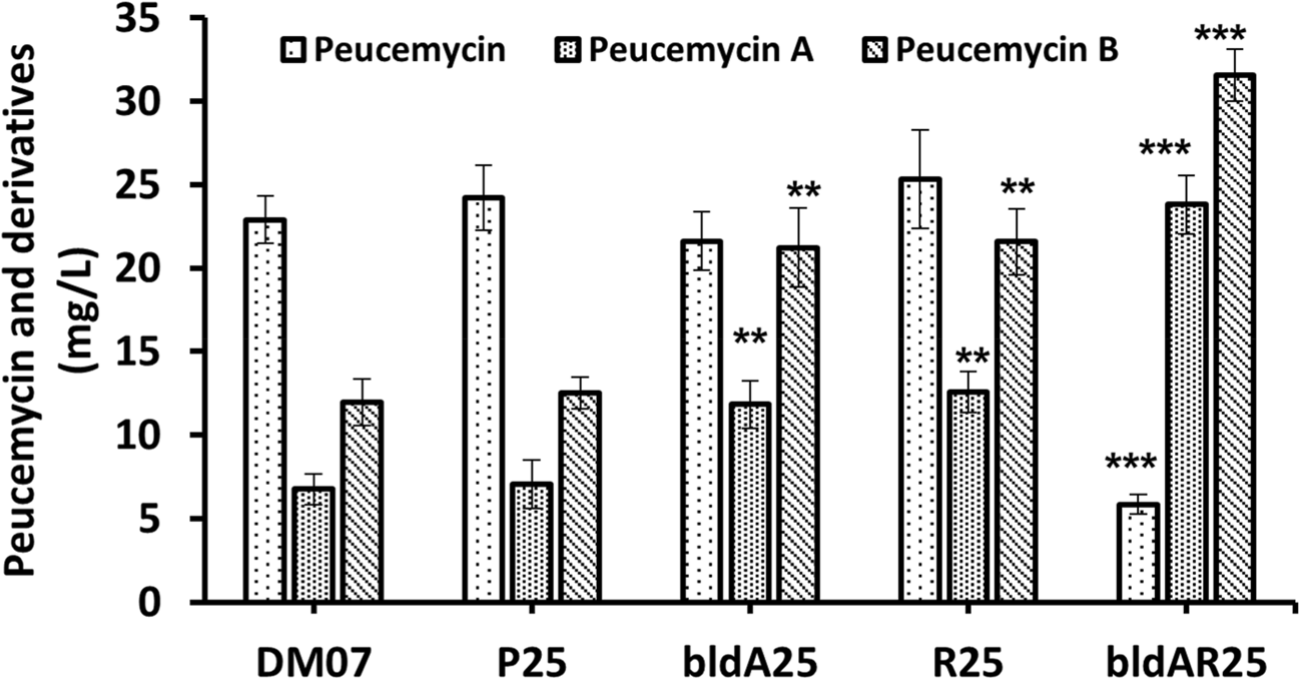
Comparison of the production of peucemycin and its derivatives. DM07, *S. peucetius* DM07; P25, *S. peucetius* P25; bldA25, *S. peucetius* bldA25; R25, *S. peucetius* R25; bldAR25, *S. peucetius* bldAR25. Standard deviation was calculated and represented as error bars from three experimental replicates for each sample. Student’s *t*-test is used to determine the *p*-values. Statistical significance: ** *p* < 0.01, *** *p* < 0.001

The impact of heterologously expressed *bldA* and overexpressed *peuR* on the production of peucemycin and its derivatives were examined in all five strains (*S. peucetius* DM07, *S. peucetius* R25, *S. peucetius* bldA25, *S. peucetius* bldAR25, and *S. peucetius* P25) (Fig. 4). There was no significant difference in the production of peucemycin or its derivatives between *S. peucetius* DM07 and *S. peucetius* P25 as evidenced by their HPLC profiles (Fig. 4). With less variation in the production of peucemycin than the wild type, *S. peucetius* bldA25 strain produced peucemycin A (11.8 mg/L) and peucemycin B (21.2 mg/L) at 1.75 and 1.77 times higher levels than the wild type, respectively. Similarly, *S. peucetius* R25 produced a 1.86 times higher level of peucemycin A (12.5 mg/L) and 1.79 times higher level of peucemycin B (21.5 mg/L) with less variation in production of peucemycin (Fig. 5). On the other hand, *S. peucetius* bldAR25 significantly altered the production of peucemycin and its derivatives when compared with the wild type (Fig. 5). This strain produced 3.52 times more peucemycin A (23.8 mg/L) and 2.63 times more peucemycin B (31.5 mg/L) than the wild type. In addition, the amount of peucemycin (5.8 mg/L) in *S. peucetius* bldAR25 was 3.92 times lower than the wild type, indicating that, in contrast to the other four strains, peucemycin was converted to its derivatives at a significant rate in this strain (Fig. 5).

**Fig. 6:**
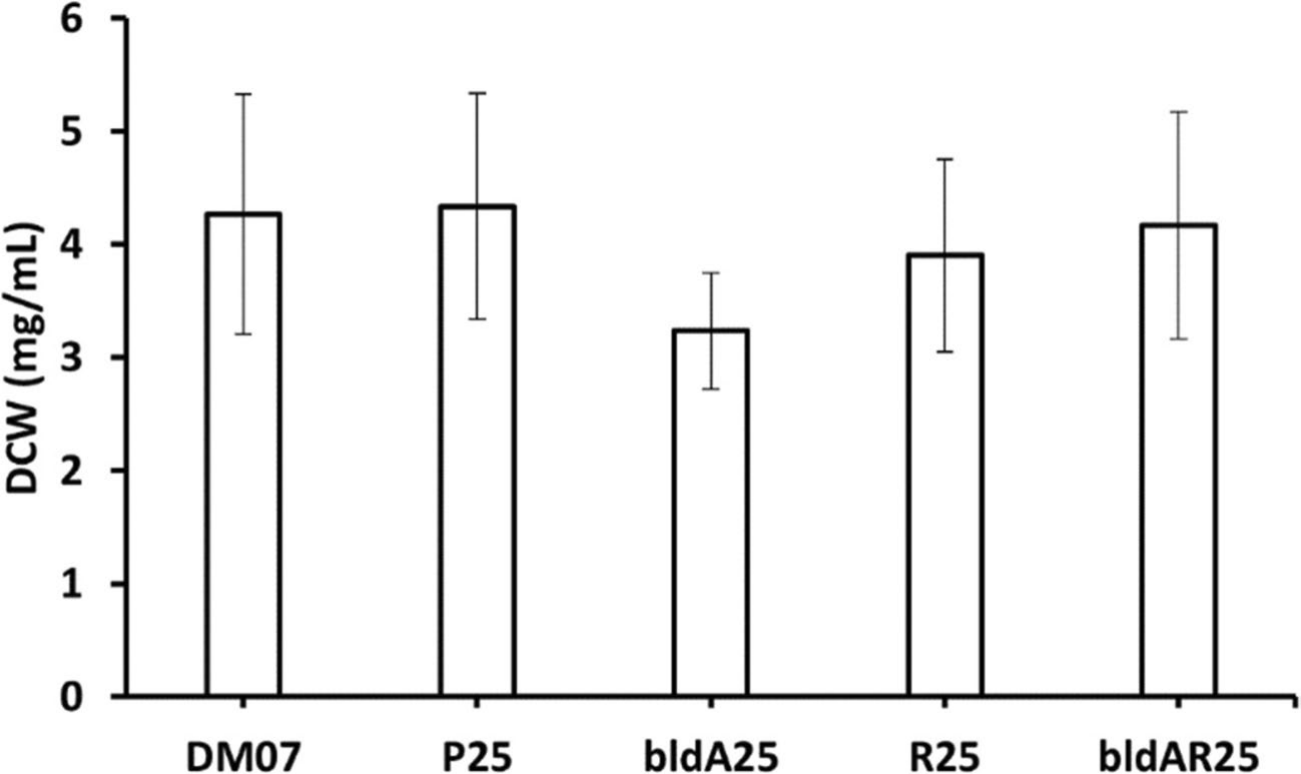
Comparison of mycelial density. DM07, *S. peucetius* DM07; P25, *S. peucetius* P25; bldA25, *S. peucetius* bldA25; R25, *S. peucetius* R25; bldAR25, *S. peucetius* bldAR25. Standard deviation was calculated and represented as error bars from three experimental replicates for each sample

Next, we sought to determine how genes affected bacterial growth. When compared to the wild type, *S. peucetius* P25 did not exhibit any significant growth differences. Among the five strains, *S. peucetius* bldA25 exhibited reduced growth (Fig. 6). On the other hand, *S. peucetius* R25 strain had a slight reduction in growth, whereas *S. peucetius* bldAR25 grew similarly to the wild type (Fig. 6).

### RT-PCR analysis of different strains

**Fig. 7:**
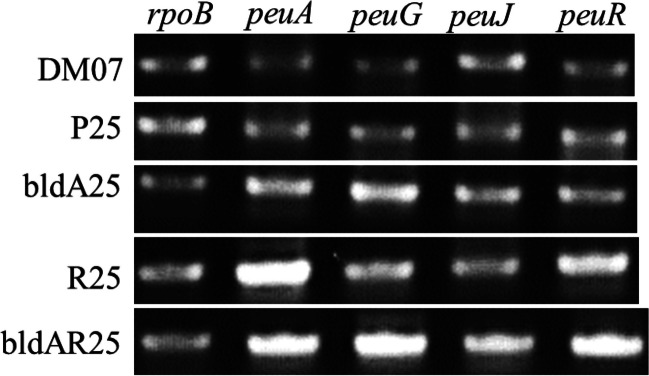
RT-PCR profiles of *rpoB*, *peuA*, *peuG*, *peuJ*, and *peuR*. DM07, *S. peucetius* DM07; P25, *S. peucetius* P25; bldA25, *S. peucetius* bldA25; R25, *S. peucetius* R25; bldAR25, *S. peucetius* bldAR25

Transcriptional levels of *peuA*, *peuG*, and *peuJ* in several strains were examined. RNA polymerase subunit gene (*rpoB*) was used as a positive internal control. *S. peucetius* DM07 and *S. peucetius* P25 showed lower transcriptional levels of *peuA*, *peuG*, and *peuJ* (Fig. 7). On the other hand, heterologous expression of *bldA* resulted in higher transcriptional levels of *peuA*, *peuG*, and *peuJ* in *S. peucetius* bldA25 as compared with the wild-type strain (Fig. 7). Similarly, overexpression of *peuR* produced a higher transcriptional level of *peuA* as seen for *S. peucetius* R25 while showing similar transcriptional levels of other genes as that of *S. peucetius* bldA25 (Fig. 7). Finally, the highest transcriptional levels of *peuG* and *peuJ* were seen in *S. peucetius* bldAR25, while similar transcriptional levels were seen for *peuA* and *peuR* as compared with *S. peucetius* R25 (Fig. 7).

## Discussion

In this work, a new peucemycin derivative (peucemycin B) was isolated, and both the pathway-specific regulator (PeuR) and global regulator (BldA) were investigated as targets for the improvement of the production of peucemycin and its derivatives. The newly identified derivative is synthesized from the hydroxylation at C-19 of peucemycin suggesting a separate hydroxylation step distinct from the C-25 hydroxylation of peucemycin A biosynthesis (Magar et al. 2023). The Peu BGC analysis provides insight into the likelihood that the PeuK is involved in the biosynthesis of peucemycin B (Magar et al. 2023).

The peucemycin B produced higher anticancer activity towards some cancer cell lines when compared to peucemycin and peucemycin A (Fig. 3 and Fig. S5) (Pham et al. 2021; Magar et al. 2023). The IC_50_ values of peucemycin B for MKN45 and NCI-H1650 cell lines were less than 100 µM, whereas those for MDA-MB-231, Hep3B, and U87MG cancer cell lines were higher than 100 µM. In comparison with some anticancer drugs that have very low IC_50_ values (Table S4), the compound is characterized as mildly active to weakly active against these cell lines (Table S3) (Jin et al. 2016; Abbasi et al. 2020; Wan et al. 2021; Choi et al. 2008; Zhan et al. 2018). The reduced solubility of the compound could be the cause of low anticancer activity. Therefore, enhancing solubility might help to improve activity.

Analysis of *peuR* revealed three TTA codons. TTA codon is regarded as a rare codon since the tRNA required for this UUA (leucine) is very rare in *Streptomyces* (Chater and Chandra 2008; Zaburannyy et al. 2009). The *S. peucetius* was found to be deficient in tRNA required for the UUA codon (Chater and Chandra 2008; Pokhrel et al. 2016). Hence, the combined effects of overexpression of *peuR* and heterologous expression of *bldA* were studied.

Similar patterns of peucemycin and its derivatives production were seen in the *S. peucetius* bldA25 and *S. peucetius* R25 strains, both of which had higher titers than the *S. peucetius* DM07 and *S. peucetius* P25 **(**Fig. 4 and Fig. 5). The results were also supported by the transcription level of some genes (Fig. 7). As predicted, overexpression of SARP gene contributed to higher production titer (Chen et al. 2015). Furthermore, the production results of *S. peucetius* DM07 and *S. peucetius* P25 suggest that a mistranslation of *peuR* mRNA may have resulted in a lower level of functional PeuR that regulates Peu BGC.

The final strain, *S. peucetius* bldAR25 produced the highest level of peucemycin derivatives (Fig. 4 and Fig. 5). The strain had the highest transcriptional levels of *peuA*, *peuG*, and *peuJ*, indicating that a functional copy of PeuR was synthesized in the presence of BldA (Fig. 7). Similarly, the results conclude that higher expression of *peuJ* is linked to the highest level of peucemycin A production. However, the gene responsible for the hydroxylation of peucemycin to peucemycin B is yet unknown and hence, the reason for the higher titer cannot be concluded from the existing results. All of these findings ultimately lead to the conclusion that the *peuR* is responsible for the regulation of Peu BGC, while BldA helps in producing a functional copy of PeuR.

In summary, we identified another peucemycin derivative and named it peucemycin B, 19-hydroxy peucemycin. Compared to peucemycin, the production of peucemycin derivatives was lower. Hence, in this study, we increased the production of peucemycin derivatives by using two regulators, a pathway-specific SARP regulator (PeuR) and a global regulator (BldA). We are now working on the gene responsible for the formation of peucemycin B.

## Supplementary Information

Below is the link to the electronic supplementary material.

Supplementary file1 (PDF 1226 KB)

## Data Availability

All data generated or analyzed during this study are included in this published article (and its supplementary information files).
