# Supplementary material for: A new peucemycin derivative and impacts of peuR and bldA on peucemycin biosynthesis in Streptomyces peucetius
Source: Appl Microbiol Biotechnol. 2024 Jan 12;108(1):107. doi: 10.1007/s00253-023-12923-4 (PMC10786969; doi:10.1007/s00253-023-12923-4)
Supplement: Supplementary file 1 — Supplementary file1 (PDF 1226 KB) [file 253_2023_12923_MOESM1_ESM.pdf]

Supplemental Information

A new peucemycin derivative and impacts of *peuR* and *bldA* on peucemycin biosynthesis in *Streptomyces peucetius*

Rubin Thapa Magar[1], Van Thuy Thi Pham[1], Purna Bahadur Poudel[1], Adzemye Fovennso Bridget[1] and Jae Kyung Sohng[1, 2 *]

[1]*Department of Life Science and Biochemical Engineering, Sun Moon University, 70 Sun Moon-ro 221, Tangjeong-myeon, Asan-si, Chungnam 31460, South Korea.*

[2]*Department of Pharmaceutical Engineering and Biotechnology, Sun Moon University, 70 Sun Moon-ro 221, Tangjeong-myeon, Asan-si, Chungnam 31460, South Korea.*

*Corresponding author: Prof. Jae Kyung Sohng

Tel: +82 (41) 530-2246

Fax: +82 (41) 530-8229

Email: sohng@sunmoon.ac.kr

List of Tables

Table S1. List of bacterial strains and plasmids used in this study.

Bacterial strains and plasmids	Description	Sources
**Bacterial Strains**		
E. coli XL1-Blue	General cloning host	Stratagene, USA
E. coli ET12567	DNA demethylating strain (dam^{-} dcm^{-} hsdS Cm^{R})	John Innes Center, UK
S. peucetius ATCC 27952	Doxorubicin and daunorubicin producer strain	ATCC
S. peucetius DM07	Doxorubicin biosynthetic gene cluster disrupted *S. peucetius* ATCC 27952	(Singh et al. 2009)
S. peucetius R25	*S. peucetius* DM07 harboring pR25 plasmid	This study
S. peucetius bldA25	*S. peucetius* DM07 harboring pbldA25 plasmid	This study
S. peucetius bldAR25	*S. peucetius* DM07 harboring pbldAR plasmid	This study
S. peucetius P25	*S. peucetius* DM07 harboring pIBR25 plasmid	This study
**Plasmids**		
pGEM®-T Easy vector	Cloning vector with Amp^{R}	Promega, USA
pIBR25	*Streptomyces* expression vector with *ermE** promoter and Tsr^{R}	(Sthapit et al. 2004)
pR25	pIBR25 expression vector harboring *peuR* gene	This study
pbldA25	pIBR25 expression vector harboring *bldA* gene	This study
pbldAR25	pIBR25 expression vector harboring *bldA-peuR* gene	This study

Table S2. List of oligonucleotides used in this study.

Primers	Sequences (5’→3’)	Restriction site	Notes
PeuR F	TCTAGACGGCTCGGAAAGTCCTTGATC	XbaI	For the amplification of *peuR*.
PeuR R	AAGCTTCTAGGACCGGCCGGCCAGCAG	HindIII	
bldA F	GGATCCAGATCTTGGAAAGCTCCGTGG	BamHI	For the amplification of *bldA*.
bldA R	TCTAGACTGCAGTCCGATCAGACAGGT	XbaI	
PeuART F	AGCCAGTAGCGGCGGCGCTGG	NA	RT-PCR primers for *peuA*
PeuART R	TCGCTGCGCCGTGACGAGGGC		
PeuGRT F	CAGGCGGCGGTAACGGCTGTG	NA	RT-PCR primers for *peuG*
PeuGRT R	CCGACGTGCGCGCTGCACTGG		
PeuJRT F	GCGCACCGCGGCGAGTTCTTC	NA	RT-PCR primers for *peuJ*
PeuJRT R	TCCGACAGTTGCGTGTGCCGG		
PeuRRT F	GGCCGCCTTCCCTTCCCGCAC	NA	RT-PCR primers for *peuR*
PeuRRT R	ACCGACCGCAAGGCGGTGGCC		
RpoB F	CAACGGCGCCAGCACTGCCCC	NA	RT-PCR primers for *rpoB*
RpoB R	CGTGGGGACGTCCTGTCCGCT		

Table S3. Anticancer (IC_{50} (µM)) potential of peucemycin B against different cell lines.

Cell lines	IC_{50} (µM)
**Cancer cell lines**	
MKN45	76.97±0.06
U87MG	150.0±0.02
MDA-MB-231	135.2±0.02
Hep3B	175.4±0.05
NCI-H1650	99.68±0.12
**Normal cell lines**	
MRC-5	325.6±1.16

Table S4. Anticancer (IC_{50} value) potential of doxorubicin and palcitaxel against different cell lines.

Cell lines	IC_{50}	References
MKN45	0.20±0.07 µM (Doxorubicin)	(Jin et al. 2016)
U87MG	5 µM (Doxorubicin)	(Abbasi et al. 2020)
MDA-MB-231	0.69 µM (Doxorubicin)	(Wan et al. 2021)
Hep3B	0.12 µM (Doxorubicin)	(Choi et al. 2008)
NCI-H1650	32.4 nM (Palcitaxel)	(Zhan et al. 2018)

List of Figures

Figure S1. Fragments analysis of peucemycin B.

Figure S2. Mycelial density of *S. peucetius* DM07 and concentration of peucemycin B on different days. Standard deviation was calculated and represented as error bars from three experimental replicates for each sample. Student's *t* -test is used to determine the *p*-values. Statistical significance: * $p < 0.05$, ** $p < 0.01$, *** $p < 0.001$.

Figure S3. 1D and 2D NMR spectra of peucemycin B. **a**) ^{1}H NMR (700 MHz, DMSO- *d*6) spectrum, **b**) ^{13}C NMR (176 MHz, DMSO-*d*6) spectrum, **c**) ^{1}H-^{1}H COSY (700 MHz, DMSO-*d*6) spectrum, **d**) ^{1}H-^{1}H ROESY (700 MHz, DMSO-*d*6) spectrum, **e**) HSQC (700 MHz, DMSO-*d*6) spectrum, and **f**) HMBC (700 MHz, DMSO-*d*6) spectrum.

Figure S4. The antibacterial activity of peucemycin and its derivatives using the disk-diffusion assay. **a**) Peucemycin B against Gram-Positive bacteria (1) positive control erythromycin (5 µg per disk), (2) negative control DMSO (2.5 µL per disk), (3) peucemycinB (11 µg per disk), and (4) peucemycinB (22 µg per disk). **b**) Against Gram-negative bacteria (1) Positive control erythromycin (5 µg per disk), (2) Peucemycin (35 µg per disk), (3) Peucemycin B (35 µg per disk), and (4) Peucemycin A (35 µg per disk).

Figure S5. Anticancer activities of peucemycin on various cancer cell lines. Standard deviation was calculated and represented as error bars from three experimental replicates for each sample.

Figure S6. Identification of rare TTA codon in *peuR*. TTA codon are highlighted in red color.

Figure S7. Agarose gel analysis for heterologous expression of *bldA* and overexpression of *peuR* of Peu BGC. **a**) PCR amplification of 873 bp *bldA*, **b**) PCR amplification of 963 bp

peuR, **c**) Ligation of the *bldA* into pGEM®-T Easy vector, **d**) Ligation of the *peuR* into pGEM®-T Easy vector, **e**) Ligation of the *bldA* into pIBR25, **f**) Ligation of *peuR* into pIBR25, and **g**) Ligation of *bldA* and *peuR* into pIBR25.

Figure S1

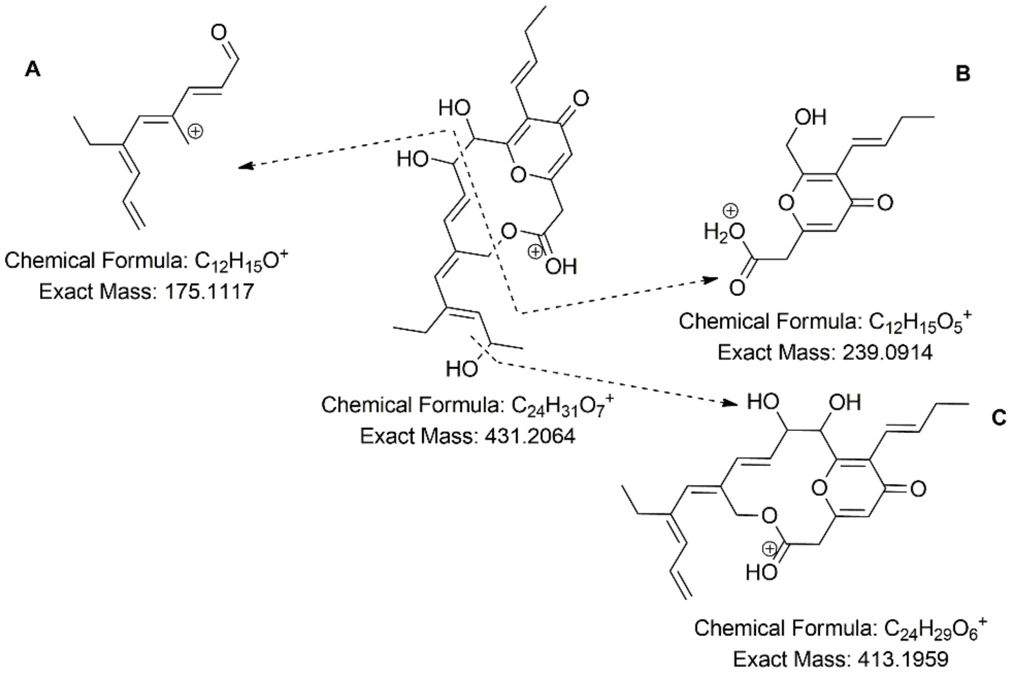
A
B
C
HO
HO
OH
O
H2O
Chemical Formula: C12H15O+
Exact Mass: 175.1117
Chemical Formula: C12H15O5+
Exact Mass: 239.0914
Chemical Formula: C24H31O7+
Exact Mass: 431.2064
Chemical Formula: C24H29O6+
Exact Mass: 413.1959

Figure S2

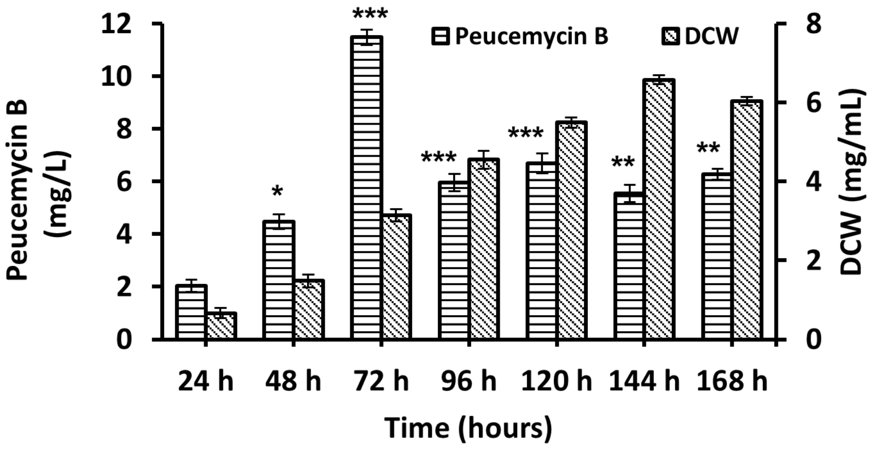

Peucemycin B
DCW
Peucemycin B (mg/L)
DCW (mg/mL)
12
10
8
6
4
2
0
8
6
4
2
0
***
*
***
***
**
**
24 h
48 h
72 h
96 h
120 h
144 h
168 h
Time (hours)

Figure S3

(a)

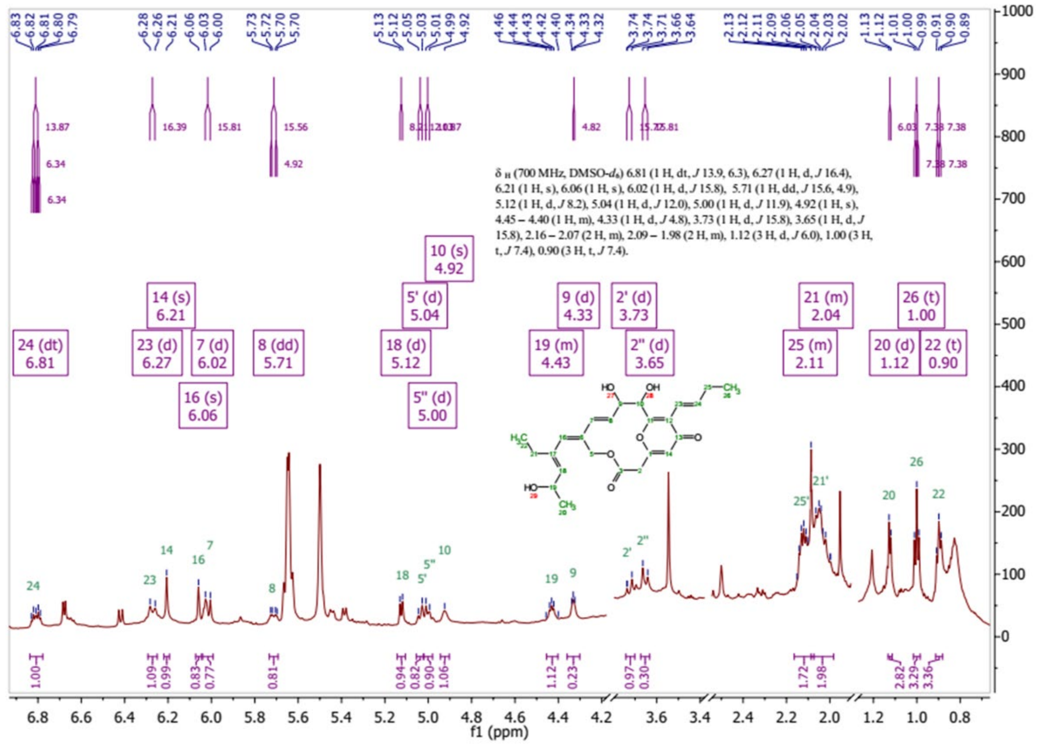

δ H (700 MHz, DMSO-d6) 6.81 (1 H, dt, J 13.9, 6.3), 6.27 (1 H, d, J 16.4), 6.21 (1 H, s), 6.06 (1 H, s), 6.02 (1 H, d, J 15.8), 5.71 (1 H, dd, J 15.6, 4.9), 5.12 (1 H, d, J 8.2), 5.04 (1 H, d, J 12.0), 5.00 (1 H, d, J 11.9), 4.92 (1 H, s), 4.45 – 4.40 (1 H, m), 4.33 (1 H, d, J 4.8), 3.73 (1 H, d, J 15.8), 3.65 (1 H, d, J 15.8), 2.16 – 2.07 (2 H, m), 2.09 – 1.98 (2 H, m), 1.12 (3 H, d, J 6.0), 1.00 (3 H, t, J 7.4), 0.90 (3 H, t, J 7.4).
24 (dt) 6.81
14 (s) 6.21
23 (d) 6.27
7 (d) 6.02
16 (s) 6.06
8 (dd) 5.71
10 (s) 4.92
5' (d) 5.04
18 (d) 5.12
5'' (d) 5.00
9 (d) 4.33
19 (m) 4.43
2' (d) 3.73
2'' (d) 3.65
21 (m) 2.04
25 (m) 2.11
26 (t) 1.00
20 (d) 1.12
22 (t) 0.90
f1 (ppm)

(b)

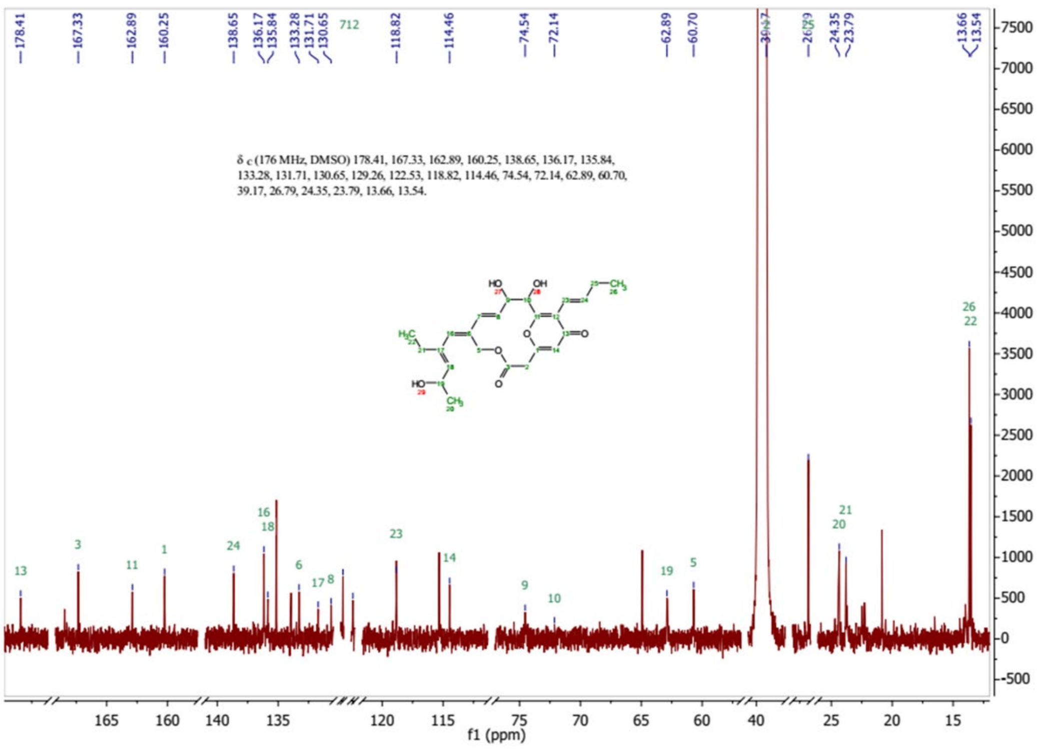

δ C (176 MHz, DMSO) 178.41, 167.33, 162.89, 160.25, 138.65, 136.17, 135.84, 133.28, 131.71, 130.65, 129.26, 122.53, 118.82, 114.46, 74.54, 72.14, 62.89, 60.70, 39.17, 26.79, 24.35, 23.79, 13.66, 13.54.
f1 (ppm)

(c)

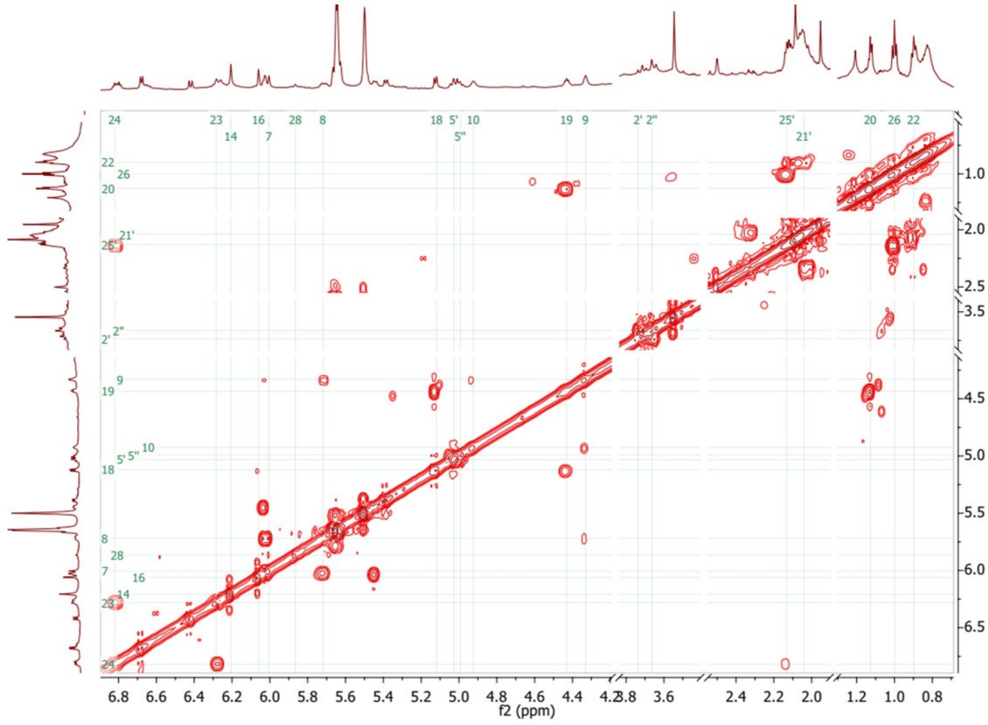
24
23
14
16
7
28
8
18
5'
5"
10
19
9
2'
2"
25'
21'
20
26
22
f1 (ppm)
f2 (ppm)

(d)

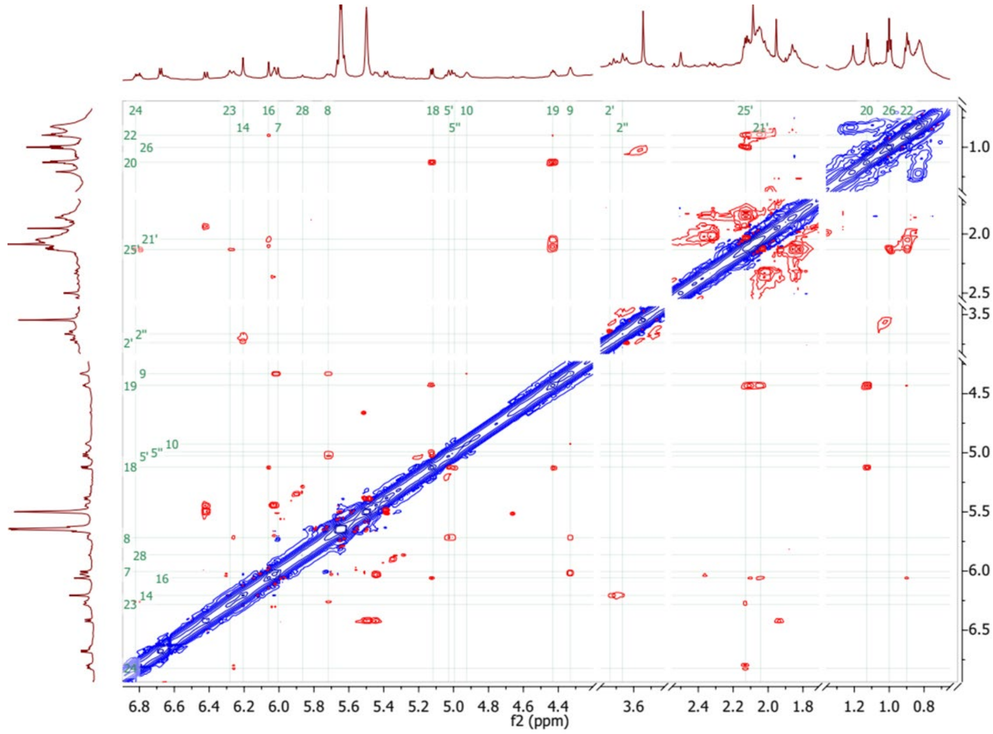
24
23
14
16
7
28
8
18
5'
5"
10
19
9
2'
2"
25'
21'
20
26
22
f1 (ppm)
f2 (ppm)

(e)

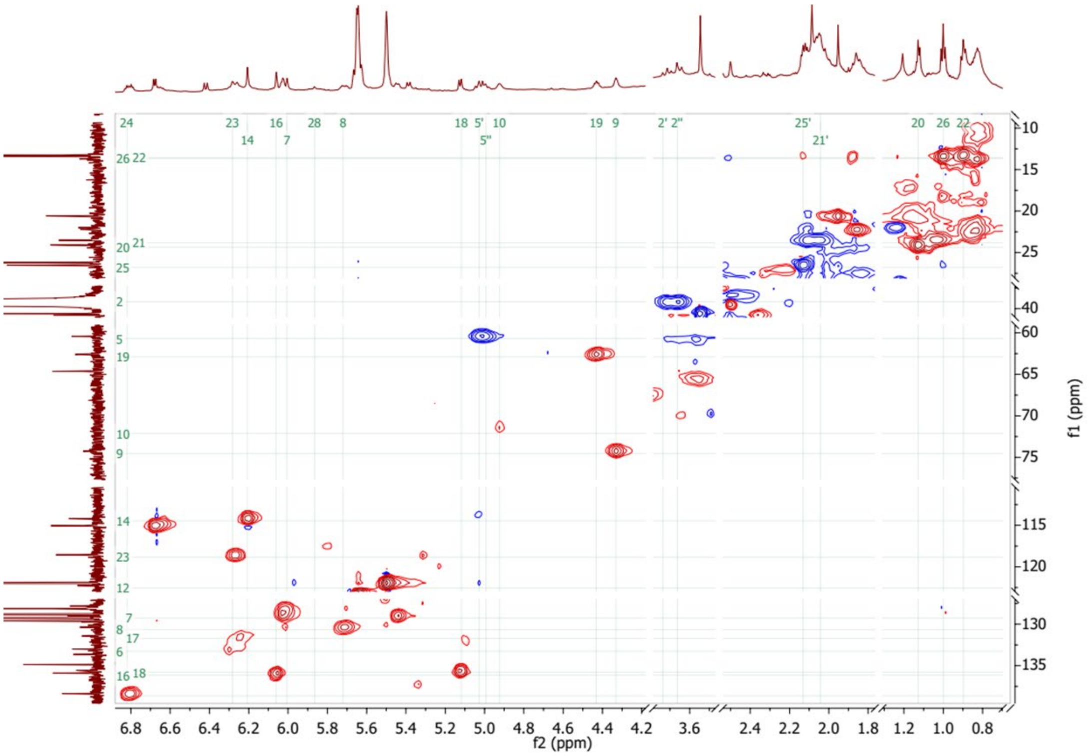

(f)

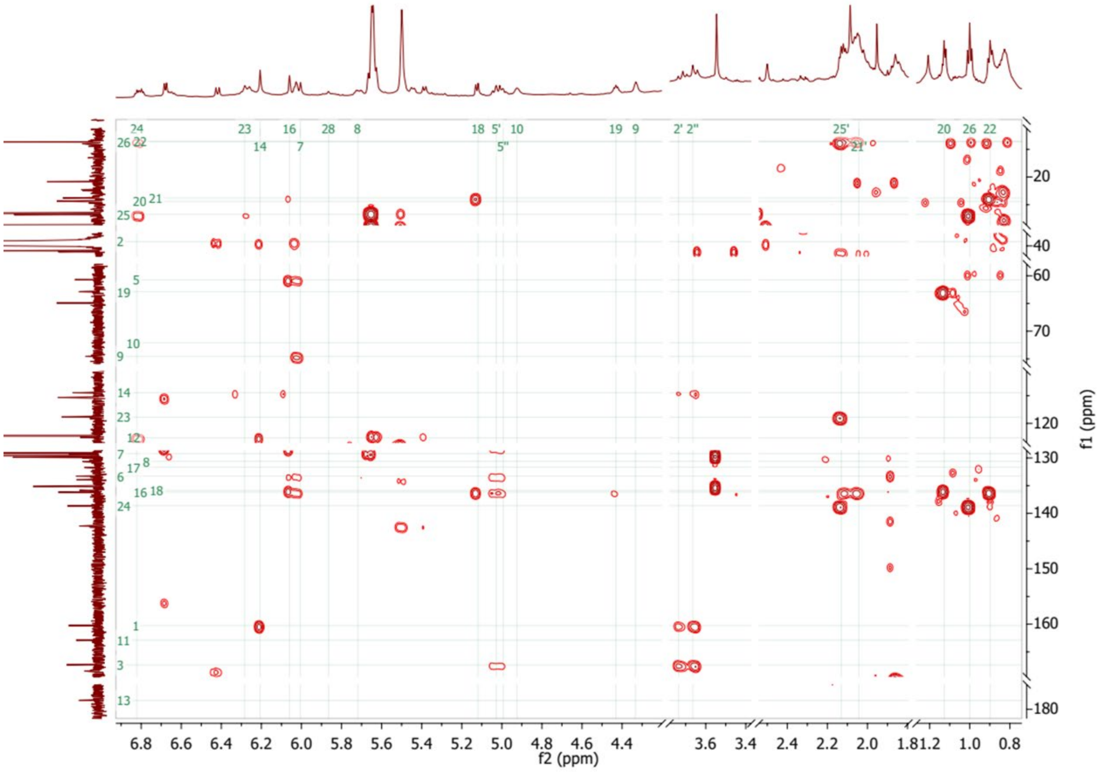

Figure S4

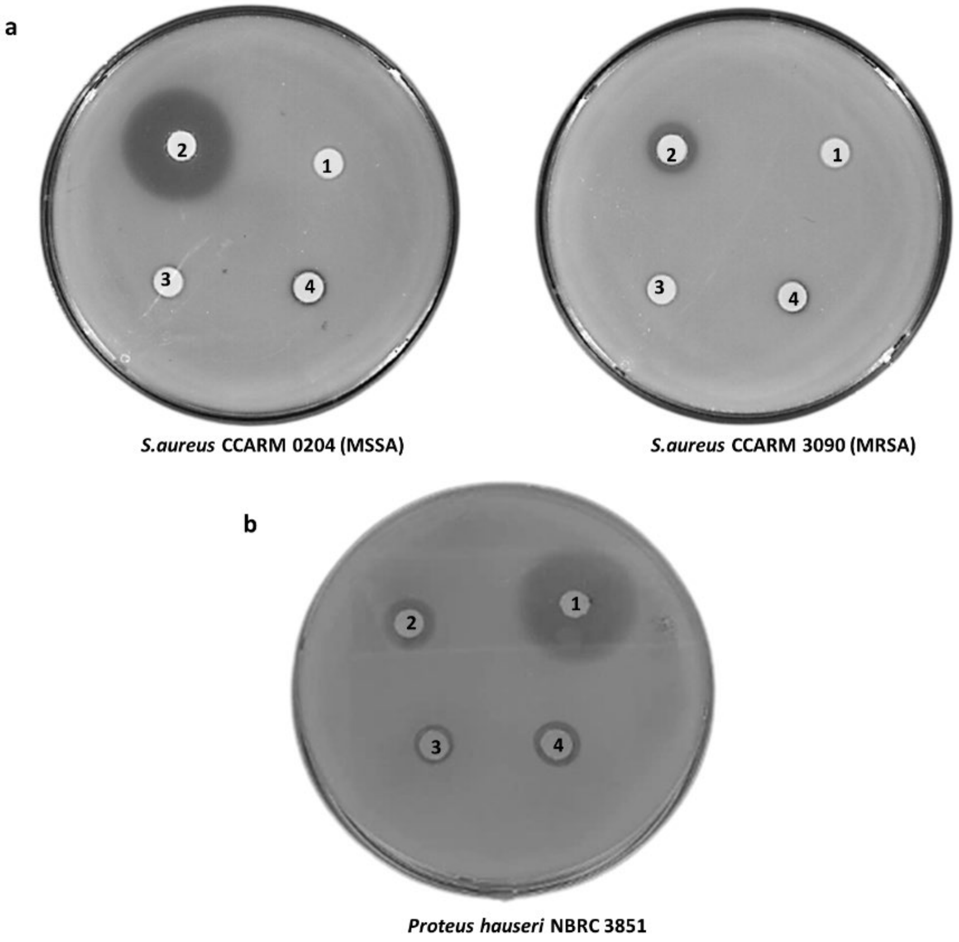
a
2
1
3
4
S.aureus CCARM 0204 (MSSA)
2
1
3
4
S.aureus CCARM 3090 (MRSA)
b
2
1
3
4
Proteus hauseri NBRC 3851

Fig. S5

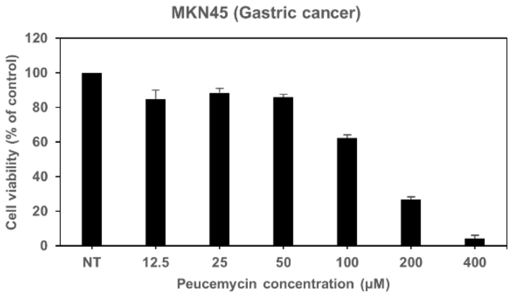

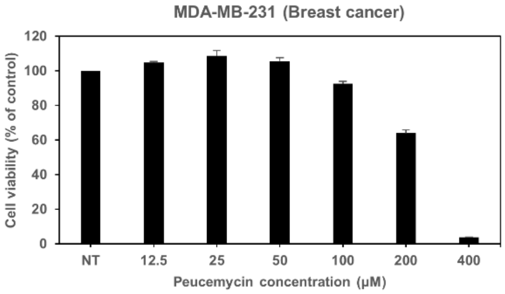

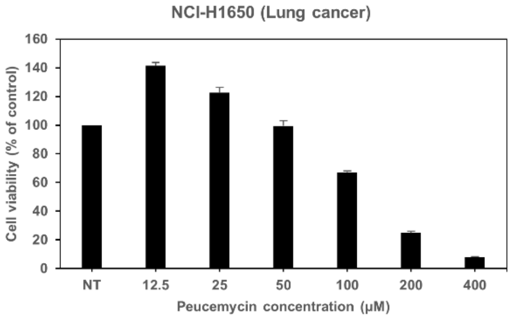

Cancer cell	MKN45	MDA-MB-231	NCI-H1650
IC_{50} (μM)	120.6 ± 0.05	223.5 ± 0.03	134.7 ± 0.12

Figure S6

```
ATGGATGTCGATGTAAGTATGTTCATCCATCACCCGTTATTCCGGCATGTCGGGTGCAGGGGGCTCTCCG
AAGGAGGGGCAATACTGATGATGCCTAATTGGATGGTACGTTCAGAGCCAAAACTCCCACCACGGGTGGG
AATACTAGGACCGTTAGAAGTCCGCTTCGCCGAGAATTCGGGATCGGGCGTTTCGGTAAATGGTATTCGC
GTTCGTGCATTACTTGCCGCGTTGACCGCGCAGCTGGGGGAGGCGATGTCGACCGAGCGAATTCTCAATG
AAGTCTGGGCCGACAACCCGCCCGCCACCGACCGCAAGGCGGTGGCCGTGGCCGTCCTGCGGCTCCGCCG
GGTCCTCGACGACCGCGATGGGCAGTGGCTGCTCACCCGCCCGTCCGGGTACGTCTTGGCCATCCCTCCG
GACCATCTTGATGCCGCAAGAGCGGAGCGCTTGGTGCGGGAAGGGAAGGCGGCCCTGACCGCAGGTGACC
CTCGCGTCGCGTCCCAGTCGCTCAGGCACGCGCTCGACCAGTGGCGCGGAGAACCATACGCCGACGCCCA
CGCCACCGCGGCGGTGGTCCGGCGCACGAGCGAATTGGAAAGCCTCAAGTCCGAGGCCGTCCAGGCCCGC
ATCGACGCCGACCTCGAACTGGGGCATCATCAGGAACTGGTCGGCGAGTTGCGCTCCCTCACCGCCGCGA
ACCCCCTGCACGAACCGCACTGGCTGCAGTTGATGCTCGCCCTGTACCGGTCGGGCAGACAGGCGGAGTC
GCTGGGCGCCTACATGGACGTCCGTCAGGCGCTCGCCGAGAAACTGGGAGTGGATCCGGGGCGGCAGCTC
CAGGAGCTGCATCTGCGGATCCTGCGCGCCGACGCCGGACTGTTGTACGGGTCGGCGACCGCGGTGTCGT
CGCAACTACTGCTGGCCGGCCGGTCCTAG
```

Figure S7

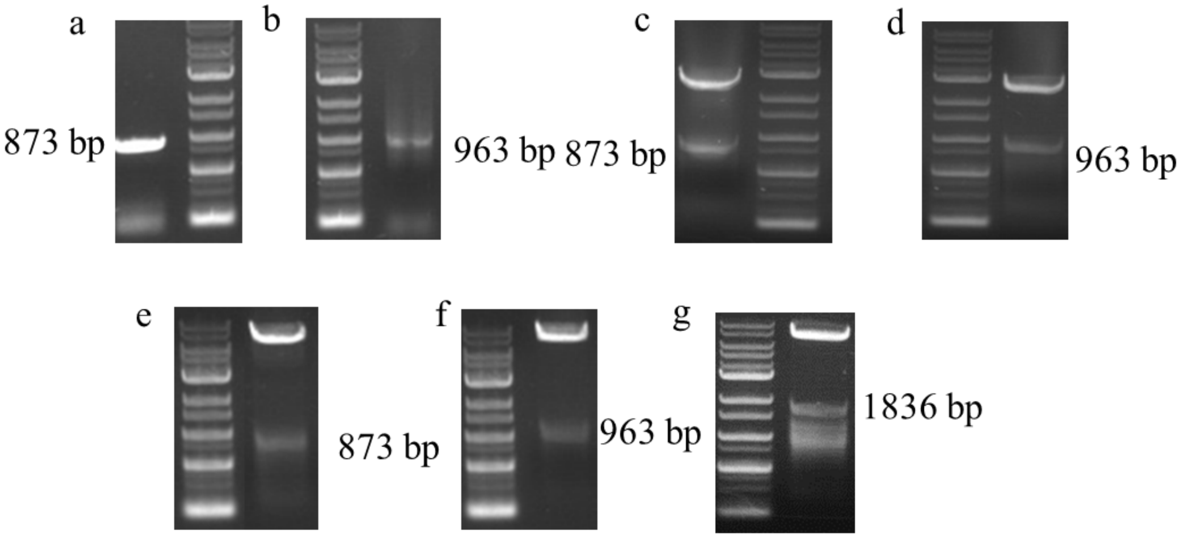

References

Abbasi A, Hajialyani M, Hosseinzadeh L, Jalilian F, Yaghmaei P, Navid SJ, Motamed H (2020) Evaluation of the cytotoxic and apoptogenic effects of cinnamaldehyde on U87MG cells alone and in combination with doxorubicin. Res Pharm Sci 15:26–35. https://doi.org/ 10.4103/1735-5362.278712

Choi J, Yip-Schneider M, Albertin F, Wiesenauer C, Wang Y, Schmidt CM (2008) The effect of doxorubicin on MEK-ERK signaling predicts its efficacy in HCC. J Surg Res 150:219–226. https://doi.org/10.1016/j.jss.2008.01.029

Jin P, Wong CC, Mei S, He X, Qian Y, Sun L (2016) MK-2206 co-treatment with 5-fluorouracil or doxorubicin enhances chemosensitivity and apoptosis in gastric cancer by attenuation of akt phosphorylation. Onco Targets Ther 9:4387–4396. https://doi.org/10.2147/OTT. S106303

Singh B, Oh TJ, Sohng JK (2009) Exploration of geosmin synthase from *Streptomyces peucetius* ATCC 27952 by deletion of doxorubicin biosynthetic gene cluster. J Ind Microbiol Biotechnol 36:1257–1265. https://doi.org/10.1007/s10295-009-0605-0

Sthapit B, Oh TJ, Lamichhane R, Liou K, Lee HC, Kim CG, Sohng JK (2004) Neocarzinostatin naphthoate synthase: an unique iterative type I PKS from neocarzinostatin producer *Streptomyces carzinostaticus*. FEBS Lett 566:201–206. https://doi.org/10.1016/j.febslet.2004.04.033

Wan X, Hou J, Liu S, Zhang Y, Li W, Zhang Y, Ding Y (2021) Estrogen receptor α mediates doxorubicin sensitivity in breast cancer cells by regulating E-Cadherin. Front Cell Dev Biol 9. https://doi.org/10.3389/fcell.2021.583572

Zhan Y, Wang K, Li Q, Zou Y, Chen B, Gong Q, Ho HI, Yin T, Zhang F, Lu Y, Wu W, Zhang

Y, Tan Y, Du B, Liu X, Xiao J (2018) The novel autophagy inhibitor alpha-hederin promoted paclitaxel cytotoxicity by increasing reactive oxygen species accumulation in non-small cell lung cancer cells. Int J Mol Sci 19:1–15. https://doi.org/10.3390/ijms19103221
